# Crystal Structure of the CTP1L Endolysin Reveals How Its Activity Is Regulated by a Secondary Translation Product*[Fn FN2]

**DOI:** 10.1074/jbc.M115.671172

**Published:** 2015-12-18

**Authors:** Matthew Dunne, Stefan Leicht, Boris Krichel, Haydyn D. T. Mertens, Andrew Thompson, Jeroen Krijgsveld, Dmitri I. Svergun, Natalia Gómez-Torres, Sonia Garde, Charlotte Uetrecht, Arjan Narbad, Melinda J. Mayer, Rob Meijers

**Affiliations:** From the ‡ European Molecular Biology Laboratory, Notkestrasse 85, 22607 Hamburg, Germany,; the § European Molecular Biology Laboratory, Meyerhofstrasse 1, 69117 Heidelberg, Germany,; the ¶ Heinrich Pette Institute, Leibniz Institute for Experimental Virology, Martinistrasse 52, 20251 Hamburg, Germany,; the ‖ Synchrotron Soleil, L'Orme des Merisiers, BP 48, Saint Aubin, 91192 Gif sur Yvette, France,; the ** Instituto Nacional de Investigación y Tecnología Agraria y Alimentaria, Departamento de Tecnología de Alimentos, Carretera de La Coruña km 7, 28040 Madrid, Spain,; the ‡‡ European XFEL GmbH, Notkestrasse 85, 22607 Hamburg, Germany, and; the §§ Institute of Food Research, Colney, Norwich NR4 7UA, United Kingdom

**Keywords:** bacteriophage, enzyme catalysis, mass spectrometry (MS), oligomerization, protein structure, Clostridia, antimicrobial, endolysin, native mass spectrometry, secondary translation

## Abstract

Bacteriophages produce endolysins, which lyse the bacterial host cell to release newly produced virions. The timing of lysis is regulated and is thought to involve the activation of a molecular switch. We present a crystal structure of the activated endolysin CTP1L that targets *Clostridium tyrobutyricum*, consisting of a complex between the full-length protein and an N-terminally truncated C-terminal cell wall binding domain (CBD). The truncated CBD is produced through an internal translation start site within the endolysin gene. Mutants affecting the internal translation site change the oligomeric state of the endolysin and reduce lytic activity. The activity can be modulated by reconstitution of the full-length endolysin-CBD complex with free CBD. The same oligomerization mechanism applies to the CD27L endolysin that targets *Clostridium difficile* and the CS74L endolysin that targets *Clostridium sporogenes*. When the CTP1L endolysin gene is introduced into the commensal bacterium *Lactococcus lactis*, the truncated CBD is also produced, showing that the alternative start codon can be used in other bacterial species. The identification of a translational switch affecting oligomerization presented here has implications for the design of effective endolysins for the treatment of bacterial infections.

## Introduction

Bacteriophages are viruses that specifically infect bacterial cells, and they can hijack the bacterial metabolism to produce new phages. To release these phages, the host cell is lysed by proteins encoded within the bacteriophage genome. The majority of double-stranded DNA bacteriophages produce two proteins that form the holin-endolysin system to accomplish host cell lysis. For the canonical holin-endolysin system, endolysins are sequestered within the cytosol, whereas the hydrophobic holins form large oligomeric lesions within the cell membrane (1). Endolysins pass into the peptidoglycan layer and degrade the cell wall, killing the bacterial host cell and releasing the bacteriophage progeny (2).

The use of recombinant endolysins as antimicrobials is potentially a promising alternative to the use of antibiotics (3). Endolysins target the structure of the bacterial cell wall, which is essential for bacterial survival. Endolysins act with high specificity, only affecting a small section of the overall microbiome, and they are less persistent than small molecule antibiotics. The risk of bacterial resistance against this class of antibactericides is therefore relatively low. An understanding of endolysin structure can lead to versions with improved stability, activity, or host range and thus improved application in therapy (4, 5). Several molecular activation mechanisms of non-canonical holin-endolysin systems have been reported. Instead of relying on holin lesion formation, endolysins containing N-terminal secretion signal peptides use the host secretion system for cell wall access. The secreted endolysin Lys-44 remains in a low activity state in the periplasm until holin-induced depolarization induces full activation of the endolysin (6, 7). Additionally, presecreted endolysins can contain a signal arrest release (SAR)^4^ anchor that also uses the host secretion system. After cell wall entry, the SAR anchor tethers the endolysins to the cytosolic membrane in an inactive state to prevent premature lysis (8). Membrane depolarization causes the SAR anchor to release the active endolysin. Reported activation mechanisms for the SAR endolysins involve repositioning of catalytic triad residues within the active site by elimination of steric hindrance (9) and disulfide isomerization mechanisms (8, 10).

Although the activation mechanisms of these presecreted endolysins have been studied in detail, little is known about the mechanisms that govern the activation of canonical endolysins. Many endolysins typically have a modular organization consisting of one or two N-terminal peptidoglycan hydrolase domains linked to a C-terminal cell wall binding domain that targets the lytic activity to a limited number of bacterial species (2). The most efficient endolysin known to date is PlyC, which targets *Streptococcus* and is assembled from two separately expressed components (11). Its structure revealed that the enzymatic component forms a non-covalent complex with eight copies of the cell wall binding component (12). However, it is not clear how this remarkable stoichiometry and the loose association between the components contribute to its efficacy.

We studied three bacteriophage endolysins that target *Clostridium* species derived from different environmental sources. The CTP1L endolysin from ΦCTP1 targets *Clostridium tyrobutyricum* (13), a food-borne bacterium associated with food spoilage in the dairy industry. The CTP1L gene encodes an N-terminal glycosyl hydrolase domain followed by a C-terminal domain (CBD) that shares a common fold with the C-terminal domains of the CD27L endolysin that targets *Clostridium difficile*, a gut pathogen that can cause debilitating and life-threatening diarrhea and colitis (14), and CS74L, which targets *Clostridium sporogenes* (15). Previously, we have shown that the CBD is involved in dimer formation that goes through an oligomeric switch affecting the endolysin activity (16). Here, we present a crystal structure of the full-length CTP1L endolysin in complex with the truncated C-terminal domain. Using high resolution tandem mass spectrometry, we show that the truncated CBD contains an N-terminal methionine. This led to the identification of a secondary translation site within the endolysin nucleotide sequence. The genetically encoded production of the truncated CBD plays an essential role in endolysin complex formation and the activity of these endolysins.

## Experimental Procedures

### 

#### 

##### Cloning, Protein Expression, and Purification

The bacteriophage nucleotide sequences of the full-length endolysins for CTP1L, CD27L, and CS74L were inserted into pET15b (Novagen), containing an N-terminal His tag and a thrombin cleavage site (13–15). Using primers CTPLCBD_FW and CTPLCBD_BW (Table 1), the C-terminal domain CTP1L(195–274) was inserted between the NcoI and XhoI restriction sites of pET21d, inserting a C-terminal His tag onto CTP1L(195–274) (CBDHis). The codon-optimized gene of CTP1L (synthetic CTP1L (sCTP1L)) (Genscript) was amplified from a pUC57 delivery plasmid using primers sCTP1L_FW and sCTP1L_BW and subcloned into the NdeI and BamHI restriction sites of the pET15b expression plasmid, the same as for the wild-type endolysin constructs. Site-directed mutants of CTP1L, sCTP1L, CD27L, and CS74L were generated following the QuikChange PCR site-directed mutagenesis protocol (Stratagene) with Phusion polymerase (New England Biolabs). Specific primer pairs used for individual mutations are listed in Table 1. CTP1L(195–274), with Val-195 altered to Met-195, was subcloned by splice overlap extension PCR to place the CTP1L CBD downstream of an N-terminally His-tagged green fluorescent protein (GFP) and a flexible linker in pET15b as described previously, using primer pair pET_F and GFPspliceCTCBD_R and primer pair CTCBDspliceGFP_F and pET_R and using CTP1L-pET15b and gfp-linker-pET15b as templates (5). All of the constructs were transformed into *Escherichia coli* BL21(DE3) (Invitrogen), and protein expression and purification were performed as described previously (16). Prior to lytic assay analysis or native MS analysis, protein samples were directly dialyzed after Ni-NTA elution into 25 mm Hepes, pH 7.4, 150 mm NaCl. Prior to crystallization or small angle x-ray scattering (SAXS) measurements, size exclusion chromatography was performed on these proteins using an S75 10/300 GL (tricorn) column (GE Healthcare) with 20 mm Hepes, pH 7.4, buffer.

**TABLE 1:** **Primers used during PCR for construct insertion into plasmids pET15b or pET21d, and primer pairs used for site-directed mutagenesis**

Primers	Sequences
**Plasmid insertion primers**	
sCTP1L_FW	5′-CGC CAT ATG AAG AAA ATC GCC GAC ATT T-3′
sCTP1L_BW	5′-CGC GGA TCC TTA TTT CAG GTT CTT GAT GTA ATC CA-3′
CTPLCBD_FW	5′-CAT GCC ATG GAA GTG GAA AAT TTA GTA GTT TA-3′
CTPLCBD_BW	5′-CCG CTC GAG TTT TAA ATT TTT AAT GTA ATC-3′
pET_F	5′-CAT CAT CAT CAC AGC AGC G-3′
pET_R	5′-GCA GCC AAC TCA GCT TCC-3′
CTCBDspliceGFP_F	5′-TGG ATC AGG TAG TGG AAT GGA AAA TTT AGT AGT TTA T-3′
GFPspliceCTCBD_R	5′-ATA AAC TAC TAA ATT TTC CAT TCC ACT ACC TGA TCC A-3′

**Mutagenesis primer pairs**	
sCTP1L_C→G_back_mutation	5′-ATC AAG TAC ATC AAG GGG GAG GAC GAA GTG GAG AAT CT-3′
	5′-AGA TTC TCC ACT TCG TCC TCC CCC TTG ATG TAC TTG AT-3′
CTP1L_G191S	5′-GAA TTT ATA AAA TAT ATT AAG TCT GAA GAT GAA GTG GAA AAT TTA-3′
	5′-TAA ATT TTC CAC TTC ATC TTC AGA CTT AAT ATA TTT TAT AAA TTC-3′
CTP1L_K190S_G191S	5′-ACT GAT GAA TTT ATA AAA TAT ATT TCT TCT GAA GAT GAA GTG GAA AAT TTA GT-3′
	5′-ACT AAA TTT TCC ACT TCA TCT TCA GAA GAA ATA TAT TTT ATA AAT TCA TCA GT-3′
CS74L_G181S	5′-ATG GAG AAT CTG GAA ACA ATA ATC AAT CTG GTA ATA AAG TGA AAG CAG TAG TA-3′
	5′-TAC TAC TGC TTT CAC TTT ATT ACC AGA TTG ATT ATT GTT TCC AGA TTC TCC AT-3′
CS74L_Q180S_G181S	5′-TGG AGA ATC TGG AAA CAA TAA TTC TTC TGG TAA TAA AGT GAA AGC AGT AGT AAT TTA T-3′
	5′-ATA AAT TAC TAC TGC TTT CAC TTT ATT ACC AGA AGA ATT ATT GTT TCC AGA TTC TCC A-3′
CD27L_E181S_G182S	5′-TGT ATT AAA TAA AAA TAT AAA TAA TTC TTC TGT TAA ACA GAT GTA CAA ACA TAC A-3′
	5′-TGT ATG TTT GTA CAT CTG TTT AAC AGA AGA ATT ATT TAT ATT TTT ATT TAA TAC A-3′

The full CTP1L coding sequence was expressed in the nisin-producing strain *Lactococcus lactis* FI5876 downstream of a signal peptide and a His_6_ tag, all under the control of the nisin A promoter PnisA (pTG262-*slpmod-His_6_-ctp1l*) to give constitutive expression and secretion (17, 18). Cloning was performed in *E. coli* MC1022, and then the construct was transformed into electrocompetent *L. lactis* FI5876 (3). Ni-NTA purification was performed on cells grown to *A*_600_ = 1.0 in 500 ml of GM17 at 30 °C, washed with 50 mm Tris-HCl, 300 mm NaCl, pH 8, and then sonicated (8 times for 15 s each). Ni-NTA eluates were pooled and concentrated using an Amicon Ultra-4 device (Millipore). Supernatants were filtered (0.22 μm) and then concentrated with Centricon Plus-70 units (3,000 nominal molecular weight limit; Millipore), and His-tagged proteins were purified and concentrated as before.

##### Crystallization and Structure Determination of the Activated CTP1L Endolysin

Protein crystals for the CTP1L heterodimer were obtained by vapor diffusion in a hanging drop setup using Limbro Plates (Hampton Research). For the crystallization drop, 1 μl of CTP1L endolysin at a concentration of 10 mg/ml was mixed with 1 μl of a mother liquor containing 5–10% PEG 8000, 20 mm Tris, pH 8.0. Crystals were harvested a few days after they appeared; transferred to a solution containing 12% PEG 8000, 20 mm Tris, pH 8.0, and 10% glycerol; and flash-frozen in liquid nitrogen. Native x-ray diffraction data were collected on the PROXIMA I beamline at the Soleil Synchrotron at 100 K using an ADSC-315 CCD detector at an x-ray energy of 12.65 keV. In addition, a highly redundant x-ray data set was collected at 6.50 keV to identify the anomalous signal from the sulfur atoms present in the CTP1L crystal. Data were processed with XDS (19) and SCALA (20). For the crystal structure of full-length CTP1L, a single crystal with space group symmetry P4_1_2_1_2 diffracted to 1.9 Å resolution (see Table 2 for data collection statistics). Molecular replacement was performed with PHASER (21) with a hybrid model built from the catalytic domains of Protein Data Bank entries 1JFX (Cellosyl) and 2NW0 (PlyB). Only one copy of the catalytic domain was found (Z score of 12.8) in the asymmetric unit. Automatic building was done with Buccanneer (22), followed by Arpwarp (23), to build two copies of the cell wall binding domain. After manual inspection with Coot (24) and the addition of TLS parameters for the individual domains, the structure was refined with Refmac5 (25) to an *R* factor of 16.4% (*R*_free_ = 20.5%). The stereochemistry of the model was verified with Molprobity (26) and contained 97.7% of the residues within the favored region of the Ramachandran plot and one residue (Glu-194, situated on the linker between the catalytic and the cell wall binding domain) in disallowed regions. The refined model was used to phase an anomalous difference density map using Phaser (27), using a cut-off for the Z score to find sites at 5.0. In this way, all sulfur atoms on the cysteine and methionine residues were identified, as well as a peak in the dimer interface between the N terminus of the truncated CBD and Val-195 of the full-length CTP1L endolysin. Omit maps were calculated by removing the ligand or residue atoms from the model, calculating the phases using Refmac5 without any refinement, and calculating an *F_o_* − *F_c_* difference map using FFT from the CCP4 package. All structure figures were created with PyMOL (PyMOL Molecular Graphics System, version 1.5.0.4, Schrodinger, LLC, New York).

**TABLE 2:** **Data collection and refinement statistics**

	CTP1L heterotetramer	CTP1L heterotetramer/sulphur single anomalous dispersion data set
**Data collection**		
Space group	P4_1_2_1_2	P4_1_2_1_2
Cell dimensions		
*a*, *b*, *c* (Å)	136.20, 136.20, 56.46	136.25, 136.25, 56.45
α, β, γ (degrees)	90, 90, 90	90, 90, 90
Wavelength (Å)	0.980	1.910
Resolution range (Å)	30.0–1.9 (2.00–1.90)*^a^*	15.0–2.5 (2.64–2.50)
No. of unique reflections	40,146	18,674
*R*_sym_	14.8 (75.4)	10.5 (76.1)
*I*/σ*I*	9.6 (2.6)	30.0 (3.2)
CC1/2	0.99 (0.70)	1.00 (0.73)
Completeness (%)	100.0 (100.0)	98.8 (95.3)
Redundancy	8.2 (8.0)	29.9 (9.0)

**Refinement**		
Resolution (Å)	30–1.90	
No. of reflections	40,146	
*R*_work_/*R*_free_	16.4 (27.3)/20.5 (32.4)	
CC_work_/CC_free_	0.96 (0.87)/0.94 (0.87)	
No. of atoms		
Protein	2782	
Ligand/ion	38	
Water	480	
*B*-Factors		
Protein	36	
Ligand/ion	52	
Water	47	
Root mean square deviations		
Bond lengths (Å)	0.01	
Bond angles (degrees)	1.1	

*^a^* Values in parentheses are for highest resolution shell.

##### SAXS Data Collection and Shape Determination

Synchrotron radiation x-ray scattering data were collected on the X33 beamline of the EMBL (DESY, Hamburg, Germany), using a 1M PILATUS pixel detector (DECTRIS, Baden-Dättwil, Switzerland) and eight frames of 15-s exposure time. Solutions of all constructs were measured at 20 °C in 20 mm Hepes buffer, pH 7.4, at protein concentrations of 0.2–4.0 mg/ml (see Table 3 for further details). Molecular masses of solutes were estimated from SAXS data by comparing the extrapolated forward scattering with that of a reference solution of bovine serum albumin. The difference curves were scaled and merged with the PRIMUS software package (28). The crystal structure of the heterotetramer of CTP1L was used to calculate a theoretical curve with CRYSOL (29). Low resolution shape envelopes for all constructs were determined using the *ab initio* bead-modeling program DAMMIF (30), using both P1 and P2 symmetry. The results of 10 multiple DAMMIF reconstructions were averaged and volume-filtered using the program DAMAVER (31) and refined in a subsequent round of DAMMIN (32) to yield representative models in agreement with the experimentally determined excluded volume and the experimental data.

**TABLE 3:** **SAXS data collection and derived parameters for CTP1L** *R_g_*, radius of gyration; *D*_max_, maximal particle dimension; *V_p_*, Porod volume; *V*_ex_, particle excluded volume.

Parameters	Values
**Data collection**	
Instrument	EMBL X33 beam line (DORIS-III, DESY, Hamburg)
Beam geometry (mm^2^)	2.0 × 0.6
Wavelength (Å)	1.54
*s* range (Å^−1^)*^a^*	0.01–0.6
Exposure time (s)	8 × 15
Concentration range (mg/ml)	0.2–4.0
Temperature (K)	293

**Structure***^b^*	
*I*(0) (relative) (from *p*(*r*))	88.6 ± 2
*R_g_* (Å) (from *p*(*r*))	40 ± 1
*I*(0) (cm^−1^) (from Guinier)	85.3 ± 0.3
*R_g_* (Å) (from Guinier)	37 ± 1
*D*_max_ (Å)	138
Porod volume estimate (Å^3^)	94,520 ± 10,000
Excluded volume estimate (Å^3^)	128,000 ± 10,000
Dry volume calculated from sequence (monomeric/dimeric) (Å^3^)	40,727/81,453

**Molecular mass determination**	
*I*(*0*) (cm^−1^) BSA (66,000 Da)	
*M*_r_ (from *I*(*0*))	65,160 ± 5000
*M*_r_ (from Porod volume (*V_p_*/1.6))	59,075 ± 5000
*M*_r_ (from excluded volume (*V*_ex_/2))	64,000 ± 5000
Calculated monomeric *M*_r_ from sequence	∼32,840

*^a^* Momentum transfer *s* = 4πsin(θ)/λ.

*^b^* Values reported for merged data sets (CTP1L: 1.0 and 4.0 mg·ml^−1^).

##### Intact Protein Sample Analysis by LC-MS

Fresh Ni-NTA-purified protein samples were dialyzed overnight into 20 mm Tris, pH 7.4, and concentrated to 2 mg/ml. Samples were acidified using 0.1% formic acid. LC-MS analysis was performed using an UltiMate 3000 RSLCnano system (Thermo Scientific) fitted with a trapping (Acclaim PepMap 100 C_18_, 3 μm, 75 μm × 20 mm) and an analytical column (Acclaim PepMap RSLC C_18_, 2 μm, 75 μm × 150 mm), coupled to a Q Exactive mass spectrometer (Thermo Scientific). The samples (around 4 ng) were loaded onto the trapping column and desalted. The proteins were eluted from the column with an acetonitrile gradient (Solvent A: water, 0.1% formic acid; solvent B: acetonitrile, 0.1% formic acid; desalting for 5 min 100% A at flow rate 6 μl/min, elution with solvent B increasing linearly from 4 to 85% in 20 min at 0.3 μl/min). Data were acquired in positive continuum mode, over a mass/charge range of 400–3000 *m*/*z* and a resolution of 70,000. Spectra across the protein chromatographic peak(s) were summed, and intact mass was calculated using the Xtract algorithm (Thermo Scientific).

Tryptic in-gel digestion was performed as described before (33) using modified porcine trypsin, sequencing grade (Promega). Full-scan MS spectra with an *m*/*z* range of 300–2000 were acquired with a resolution of 70,000. The most intense ions (up to 15) from the full-scan MS were selected for fragmentation. Tandem MS (MS/MS) was carried out with a resolution of 17,500 with a fixed first mass at 100 *m*/*z* and normalized collision energy of 25 V. The raw data were processed using MaxQuant (34), and MS/MS spectra were searched using the MASCOT search engine (version 2.2.07, Matrix Science).

##### Native Mass Spectrometry

Purified proteins were buffer-exchanged prior to MS analysis to 150 mm ammonium acetate (99.99% purity; Sigma-Aldrich), pH 7.4, via centrifugal filter units at 15,600 × *g* (Vivaspin 500, molecular weight cut-off 5000; Sartorius) or dialysis devices (Slide-A-Lyzer 100 μl, 3500 molecular weight cut-off; Thermo Scientific) at 4 °C. Titrations of the CBD were performed with a 10 μm concentration of the full-length protein. Native MS was carried out on a QToF 2 (Waters (Cheshire East, UK) and MS Vision (Almere, The Netherlands)) modified for high mass experiments (35) with a nano-electrospray ionization (nano-ESI) source in positive ion mode. The gas pressures were 10 millibars in the source region and 1.1 × 10^−2^ millibars of xenon (purity 5.0) or 1.5 × 10^−2^ millibars of argon in the collision cell (36, 37). Borosilicate capillaries (1.2-mm inner diameter, 0.68-mm outer diameter, with filament; World Precision Instruments) were processed into closed capillaries for ESI in a two-step program with a micropipette puller (P-1000, Sutter Instruments) using a squared box filament (2.5 × 2.5 mm; Sutter Instruments) and subsequently gold-coated using a sputter coater (40 mA, 200 s, tooling factor of 2.3, and end bleed vacuum of 8 × 10^−2^ millibars of argon, Q150R; Quorum Technologies Ltd). Capillaries were opened on the sample cone of the instrument. Mass spectra were recorded with applied voltages for capillary, cone, and collision of 1.3–1.35 kV, 130–140 V, and 10–20 V, respectively, optimized for minimal complex dissociation. MS/MS collision voltages were ramped from 10 to 130 V to confirm and assign protein complex species. MassLynx software (Waters) and Massign (38) were used to assign the peak series to protein species and to determine the mass. Masses as well as ratios of protein species were determined from at least three independent measurements, and errors given correspond to the S.D.

##### Analysis of Cell Binding and Lysis

Assessments of lytic and binding activity were performed using Ni-NTA-purified protein as described previously, using buffer or Ni-NTA-purified GFP linker protein, respectively, as negative controls (5). For fluorescence microscopy, cells were viewed with a ×100 magnification oil immersion lens.

## Results

### 

#### 

##### The Crystal Structure of Endolysin CTP1L Contains a Truncated C-terminal Domain

**FIGURE 1.**
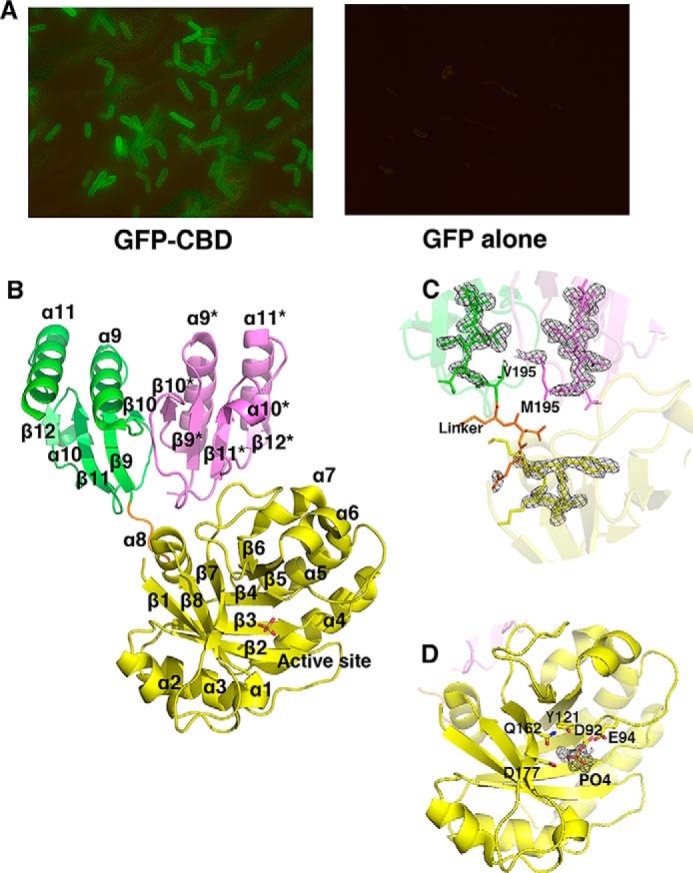
*A*, fluorescence microscopy images of binding of GFP-CBD to cells of *C. tyrobutyricum* (*left*, ×100 magnification lens) and GFP alone (*right*, ×40 magnification lens) showing no binding. *B*, *ribbon diagram* of the CTP1L endolysin in activated form, consisting of a complex between one copy of the full-length endolysin and one copy of the truncated C-terminal domain (*tCBD*) in *pink*. The EAD is *colored* in *yellow*, and the active site is *marked* with the phosphate ion shown as *sticks*. The CBD that is part of the full-length endolysin is *colored green*. The secondary structure elements are *labeled*, and the linker between domains is *colored orange. C*, *enlargement* of the interface between the CBD, the truncated CBD, and the EAD, showing an electron density *F_o_* − *F_c_* omit map at 3σ contour level. The residue Val-195 at the start of the CBD and Met-195 at the start of the truncated CBD are *highlighted. D*, *enlargement* of the active site area, showing the residues that coordinate the phosphate ion in sticks. An *F_o_* − *F_c_* omit map of the phosphate ion is shown at 3σ contour level.

CTP1L protein that is expressed in *E. coli* using the native bacteriophage oligonucleotide sequence reveals the presence of two species of different molecular weight on an SDS-polyacrylamide gel when purified (16). Besides the full-length endolysin, there is a comparable amount of truncated CBD produced. To verify whether the CBD was a cell wall binding domain, a construct was made of the CBD domain alone with an N-terminal GFP attached. When purified GFP-CBD was mixed with *C. tyrobutyricum* cells, binding of the proteins to the cells was observed by fluorescence microscopy (Fig. 1*A*). As a control, GFP alone did not show any binding. This indicates that the CBD can bind to the cell wall of *C. tyrobutyricum* even when applied externally.

The crystal structure of CTP1L was determined by molecular replacement using the catalytic domain of an endo-*N*-acetylmuramidase cellosyl from *Streptomyces coelicolor* (39) (Protein Data Bank code 1JFX) as a search model. The structure was refined to a resolution of 1.9 Å (Table 2), and it contains one copy of a full-length CTP1L endolysin as well as a copy of the truncated CBD in the asymmetric unit (Fig. 1, *B* and *C*). The full-length CTP1L endolysin consists of the enzymatically active domain (EAD) (residues 1–190) and a CBD (residues 195–274) connected by a linker of four residues (Fig. 1*B*). The electron density for the EAD and the CBD are clearly defined, but the linker between the domains shows weak electron density (Fig. 1*C*). The EAD consists of a (β/α)_5_β_3_ barrel, where strand β6 is directly connected to strand β7, and strand β8 runs anti-parallel to the other β strands. This fold is similar to cellosyl (39) and the catalytic domain of endolysin Psm (40). The active site of the CTP1L catalytic domain contains a phosphate ion, surrounded by the catalytic residues Asp-92, Glu-94, Tyr-121, and Asp-177. These residues are conserved among CTP1L, cellosyl, and PlyB, and the active site is situated opposite the CBD (Fig. 1*D*).

The truncated CBD is closely associated with the CBD of the intact endolysin, in an arrangement that is similar to the CBDs reported earlier for the crystal structure of the CTP1L mutant V195P (16). The two CBDs are arranged side-by-side, and the buried surface area covered by the truncated CBD is 2560 Å^2^. The largest portion (70%) of the buried surface area is covered by the CBD of the intact enzyme, but the contribution from the catalytic domain and the linker together is also substantial. Although several residues from the enzymatically active domain are involved in interactions with the truncated CBD, none form hydrogen bonds. In contrast, there is an extensive hydrogen-bonding network between the truncated CBD and the CBD of the full-length endolysin.

##### CTP1L Forms Predominantly Heterotetramers in Solution

Further oligomerization occurs through a head-on dimer interface, so that the CBD of the intact endolysin forms a dimer with the CBD of another intact endolysin. In parallel, the truncated CBD forms a head-on dimer with another truncated CBD. The head-on oligomerization mode has also been observed previously for a truncated CD27L CBD domain as well as the CTP1L V195P mutant (16). The close association of the head-on and side-by-side dimers creates a heterotetramer assembly that buries a surface area of 8410 Å^2^, suggesting that the heterotetramer is the predominant oligomeric state of CTP1L present in solution. The arrangement of the CBDs obeys D2 symmetry, and the heterodimer follows C2 symmetry.

Using size exclusion chromatography, the CTP1L endolysin appears as a single oligomeric species, in contrast to CD27L, which occurs as a complex mixture of oligomers containing both the full-length endolysin and the truncated CBD (16). To determine the composition of the endolysin in solution, SAXS was performed on the purified CTP1L endolysin.

**FIGURE 2.**
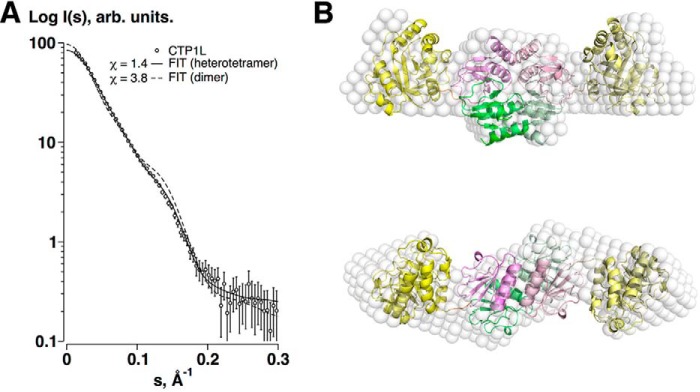
*A*, SAXS data of the CTP1L endolysin (*circles*), and the fits of the heterotetrameric (*solid line*) and dimeric (*broken line*) models. *B*, superposition of the *ab initio* dummy atom model reconstructed from the SAXS data for CTP1L (*white spheres*) and the crystal structure of the heterotetramer of CTP1L (*schematic*), with the CBDs located centrally at the oligomer interface and the EADs at the periphery. The *view* is along the 2-fold symmetry axis at the *top* and is *rotated* by 90º in the *bottom panel*.

The scattering data for CTP1L are consistent with that of a heterotetramer configuration (Table 3 and Fig. 2*A*). Envelope reconstruction with DAMMIF (30) results in an elongated shape with a prominent central core (Fig. 2*B*), and comparison with a theoretical curve derived from the heterotetramer CTP1L crystal structure fits the data well.

##### The Truncated CBD Contains an N-terminal Methionine

**FIGURE 3.**
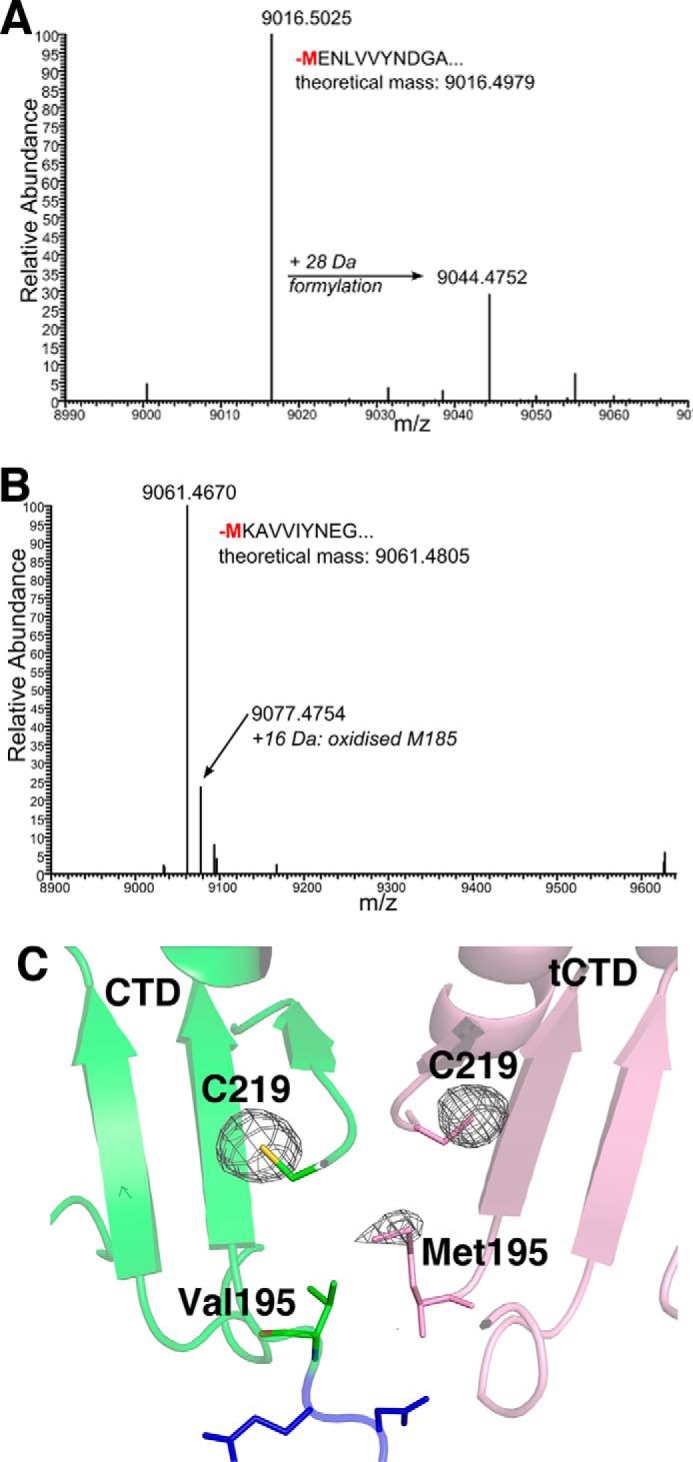
*A*, mass over charge (*m*/*z*) spectrum of the liquid chromatography fraction from wild-type CTP1L, showing the experimental mass of the truncated CBD containing a methionine. *B*, same spectrum for wild-type CS74L. *C*, anomalous difference map of the CTP1L crystal structure near the sulfur edge, showing the presence of a sulfur atom at the N terminus of the truncated CBD. The peaks of the phased anomalous difference density map are shown as a *mesh* at a contour level of 4σ.

To determine how the truncated CBDs of the CTP1L and CS74L endolysins studied here are produced, we performed high resolution liquid chromatography coupled to electrospray ionization mass spectrometry (LC-ESI-MS). Previously, we used this method to determine the chemical composition of the truncated CBD from CD27L, and we found that the experimental mass exactly matched a polypeptide starting at Met-186 (16). However, when LC-ESI-MS was performed on the truncated CBD from CTP1L, the experimental mass surprisingly did not exactly correspond to the polypeptide observed in the crystal structure (Fig. 3*A*). The N-terminal residue in the crystal structure of CTP1L is at position 195, which codes a valine, but the experimental mass was 32 Da higher than expected. Further analysis by tryptic digestion followed by peptide identification with MS/MS showed that the N terminus of the truncated CBD contained a methionine. A similar observation was made for CS74L, where the N-terminal peptide starting at position 185 contained a methionine instead of a valine (Fig. 3*B*).

The presence of a sulfur atom at the N terminus of the truncated CBD was confirmed by an x-ray diffraction experiment on a crystal of the CTP1L heterotetramer complex, performed near the sulfur edge (6.5 keV). An anomalous dispersion map revealed the presence of a sulfur site situated between Val-195 of the full-length endolysin and the N-terminal Val-195 of the truncated CBD (Fig. 3*C*). Additional mass spectrometry analysis using tryptic digestion linked with LC-MS/MS on the truncated CTP1L and CS74L CBDs after SDS-PAGE analysis and excision confirmed that the N-terminal amino acid of the CBDs was a methionine.

##### Identification of an Internal Ribosomal Binding Site and Alternative Start Codon

**FIGURE 4.**
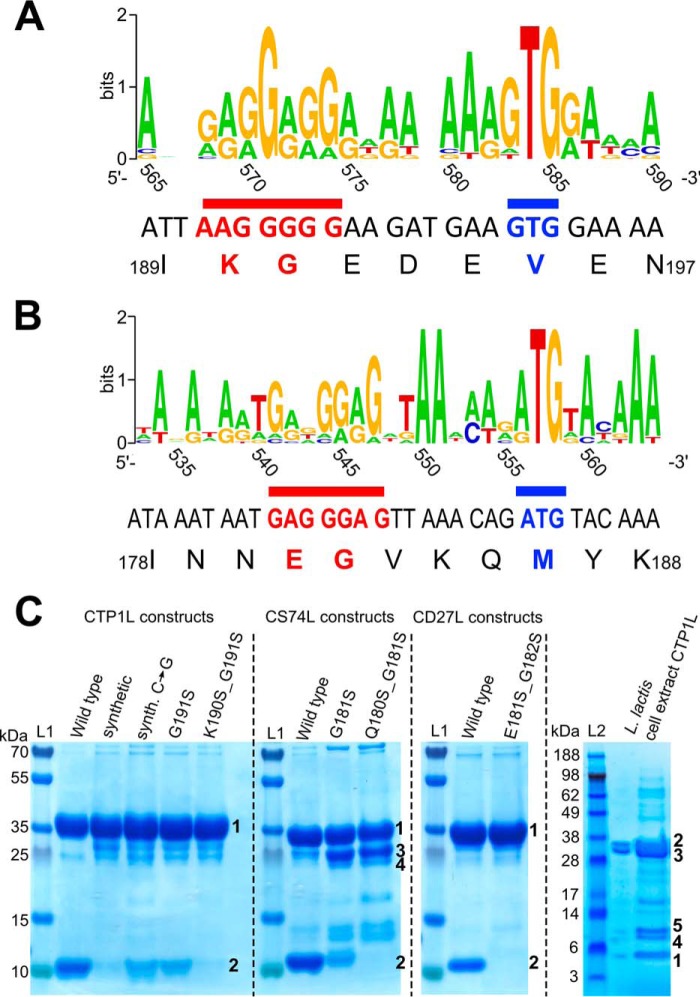
*A*, sequence logo created with Weblogo (49) showing the linker region of CTP1L with nucleotides *numbered* according to the CTP1L nucleotide sequence based on a sequence alignment of CTP1L-related lysins as presented by Dunne *et al.* (16). The Shine-Dalgarno region is *underlined* in *red*, and the start codon is *underlined* in *blue. B*, sequence logo showing the linker region of CD27L with nucleotides numbered according to the CD27L nucleotide sequence. The Shine-Dalgarno region is *underlined* in *red*, and the start codon is *underlined* in *blue. C*, SDS-PAGE shows the effect of mutations in the ribosomal binding site of endolysins CTP1L, CD27L, and CS74L on the expression of the truncated CBD. The wild-type protein produced from the wild-type bacteriophage nucleotide sequence is compared with a codon-optimized (synthetic) gene for CTP1L. A silent mutation of CTP1L is shown (*Synth C* → *G*), which restores the RBS region from the codon-optimized gene sequence to the wild-type sequence. Mutants of the glycine (GGG) that forms part of the RBS in CTP1L and CS74L to serine (TCT) do not abolish expression, but the RBSKO mutations (K190S/G191S for CTP1L, E180S/G181S for CS74L, and E181S/G182S for CD27L) reduce expression of the truncated domain drastically. *Lane L*, marker. *Bands marked* as *1* have been identified as full-length protein; *bands marked* as *2* have been identified to contain an N-terminal methionine and are the product of secondary translation. *Bands marked* with *3* and *4* occur in CS74L constructs where the secondary translation site has been compromised, and these bands have been identified by peptide fingerprinting as degradation products of full-length CS74L. The *far right panel* shows pooled concentrated eluates of N-terminally His-tagged CTP1L expressed in *L. lactis*. Excision of bands and analysis by tryptic digest and MALDI-ToF-MS showed that this *band 1* contained 100% of the peptides expected from the translation product of the alternative start site (Met-195) with no incidence of Val-195. The *larger bands* (*2* and *3*) contained peptides of the full-length sequence, including Val-195, whereas *bands 4* and *5* both contained peptides matching the secondary translation product in addition to selected peptides from the full-length lysin sequence.

The consistent identification of methionine as the N-terminal residue of the truncated CBDs indicated that the truncated CBD is a secondary translation product of the endolysin mRNA. A sequence alignment on the nucleotide level of CTP1L and CS74L with other related endolysins shows the presence of a putative Shine-Dalgarno region prior to the alternative start codon GTG (Fig. 4*A*). Based on the alignment, the putative Shine-Dalgarno sequence in the linker region of CTP1L and CS74L is AAGGGGG. For most CTP1L-like endolysins, the putative Shine-Dalgarno region contains GGAGG. For the CTP1L and CS74L endolysins, the second and third nucleotides (TG) of the start codon are conserved among related lysins (Fig. 4*A*). There is also a putative Shine-Dalgarno sequence for the CD27L endolysin upstream of the secondary ATG start codon encoded as GAGGGAG (Fig. 4*B*). Strikingly, the canonical ATG start codon is present for all but one of the CD27L-related endolysins.

The nucleotide sequence used during *E. coli* expression was derived from the original bacteriophage DNA. To test whether the wild-type sequence encoded a secondary translation site, a synonymous codon-optimized gene was engineered for CTP1L and inserted into the pET15b *E. coli* expression vector that was also used for the wild-type CTP1L (supplemental Fig. S1). Expression of the synthetic gene product resulted in a substantial reduction of the truncated CBD (Fig. 4*C*), suggesting that the production of the truncated CBD is related to the nucleotide sequence of the endolysin gene.

To investigate whether this phenomenon was an artifact of expression in *E. coli*, an *L. lactis* strain designed for continuous endolysin production and secretion was analyzed. SDS-PAGE of Ni-NTA-purified proteins from cell extracts of *L. lactis* expressing pTG262-*slpmod-His_6_-ctp1l* showed a faint band that migrated to the same size as the truncated CBD expressed from *E. coli*, in addition to the full lysin (Fig. 4*C*). After concentration, mass spectrometry of tryptic digests from this excised band confirmed the sequence as the truncated CBD with a Met residue at the start and no evidence of Val-195. The lysin was also seen in the supernatant, but concentrations were not high enough to determine whether the truncated CBD was present.

##### Knock-out of the Secondary Translation Site Affects Oligomerization

In the codon-optimized gene, the original Shine-Dalgarno sequence is mutated in one nucleotide position to AAGGGCG (mutation is underlined), which may have led to the substantial reduction in the translation of the truncated CBD. In addition, there is a silent mutation between the putative Shine-Dalgarno sequence and the alternative start codon in Glu-192 from AAG to AGG. To test whether these modifications affect CBD production, a single synonymous silent back-mutation in the synthetic gene was made. The production of truncated CBD was enhanced for the C → G back mutation, because the Gly_GGG_-191 mutant produces more truncated CBD than the Gly_GGC_-191 mutant (Fig. 4*C*).

The codon-optimized synonymously mutated CTP1L does not completely abolish the production of the truncated CBD. The residues Lys-190 and Gly-191 that encode the putative ribosomal binding site were therefore mutated in the wild-type CTP1L nucleotide sequence to serines (K190S/G191S, from now on called ribosomal binding site knock-out, RBSKO). These residues are situated at the start of the linker region between the catalytic and the C-terminal domain and do not interact with either domain. The RBSKO mutant modifies the ribosomal binding site substantially and inhibits the production of the truncated CBD completely to the extent that it could not be detected in a highly concentrated sample by mass spectrometry. The modification of the ribosomal binding sites was further extended to CS74L and CD27L (Fig. 4*C*), showing for all three endolysins that a double mutation at the beginning of the linker abolished secondary translation of the CBD.

##### CTP1L Endolysin Activity Is Modulated by the Presence of Truncated CBD

**FIGURE 5.**
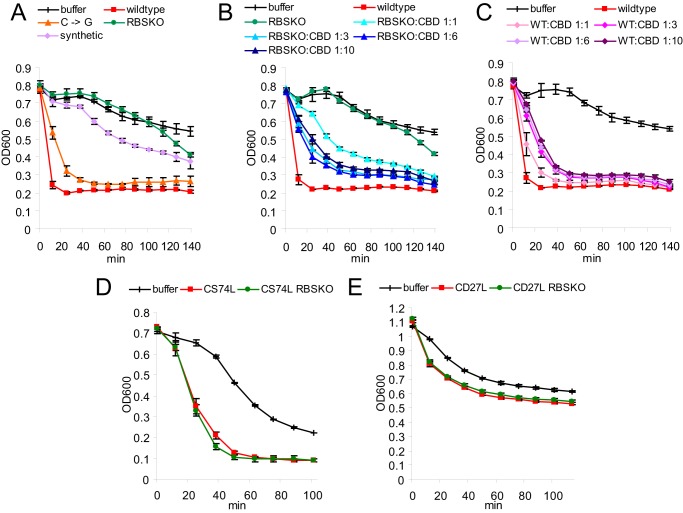
*A–C*, lysis assays performed on *C. tyrobutyricum* using CTP1L wild type protein (0.1 μm) as well as mutants that silence truncated CBD expression. *B*, the addition of increasing ratios of CBDHis to 0.1 μm K190S/G191S/RBSKO. *C*, repression of wild-type CTP1L activity with CBDHis. *D* and *E*, lysis assays performed on *C. sporogenes* using CS74L (*D*) and *C. difficile* using CD27L (*E*) wild type protein, as well as RBSKO mutants (concentration 0.1 μm) show no effect of the secondary translation product on lytic activity. The decrease in optical density over time is a measure of lysis. Results represent the mean of duplicates ± S.D.

The oligomerization of the CTP1L endolysin has been shown to affect lysis efficiency (16). Recombinant CTP1L endolysin produced from the codon-optimized gene was applied to *C. tyrobutyricum* cells, and reduction in optical density was followed as an indication of lytic activity (Fig. 5*A*). In comparison with recombinant CTP1L produced from the wild-type nucleotide sequence, there is a clear reduction in lysis activity. The codon-optimized CTP1L, which has severely reduced levels of the truncated CBD, takes significantly longer to reach the same level of optical density as the wild-type CTP1L. In contrast, the silent mutation (C → G) that reinstates the ribosomal binding site prior to the alternative start codon partially restores *C. tyrobutyricum* lysis. However, the RBSKO mutant produces a further loss of activity with no lysis seen for the first 2 h of incubation. At increased endolysin concentrations, all mutants showed greater lytic activity, but the relative efficiencies remained the same.

A construct was produced consisting of the CBD of CTP1L with Val-195 changed to Met-195 as the start codon, with a His tag attached to the C terminus (CBDHis). The purified protein was mixed with the CTP1L endolysin RBSKO mutant. It was shown that lysis efficacy could be modified with increasing concentrations of CBDHis (Fig. 5*B*). Conversely, the addition of CBDHis to the wild-type enzyme was detrimental to lytic efficiency (Fig. 5*C*). Further addition of CBDHis to more concentrated (1 μm) RBSKO-modified endolysin was less effective in improving activity, suggesting that once there is an excess of CBD, the lysis efficiency is reduced by the presence of free CBDHis. This may be due to saturation of binding sites or to aggregation. These experiments all indicate that the truncated CBD is an aid to lysis rather than a requirement and therefore is not effective when the enzyme is applied *in vitro* at higher concentrations. However, it probably plays a role in physiological conditions, where low concentrations of lysin attack the cell wall from the inside. Similar alterations of the RBS in CD27L and CS74L, which do not require their CBD for activity, had no effect on lytic activity (Fig. 5, *D* and *E*).

##### Native Mass Spectrometry Relates Oligomeric States to Lysis Activity

**FIGURE 6.**
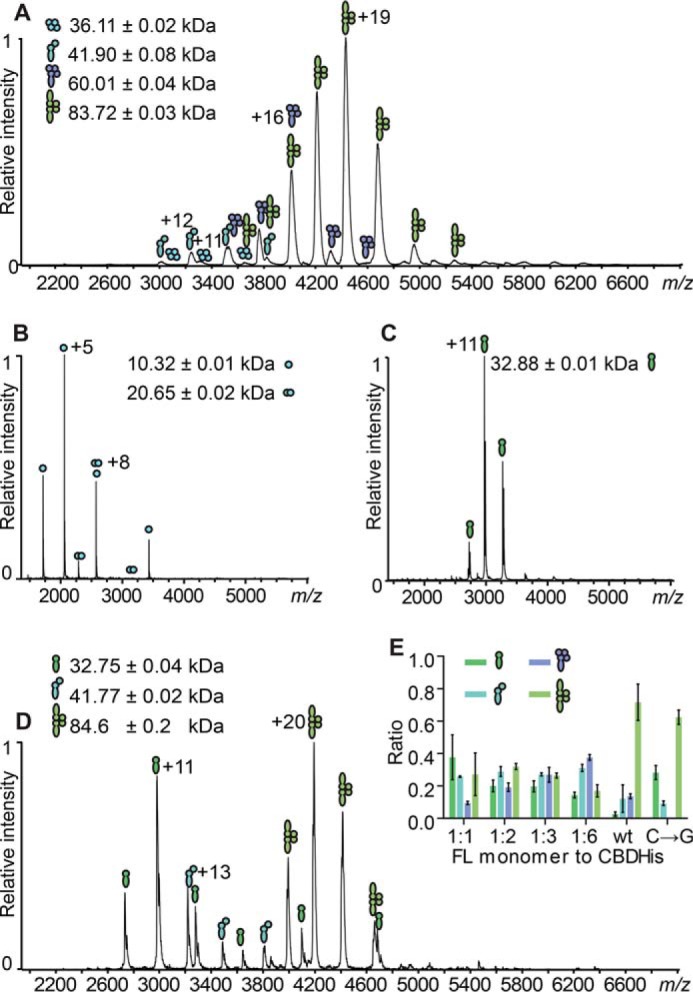
*A*, ESI-MS analysis of wild type CTP1L. Indicated are the charge state distributions of different complexes formed by the non-covalent association of the CBD with the full-length CTP1L in the native state. Indicated are symbols representing the various complexes formed: CBD tetramer, CBD/FLCTP1L heterodimer, 1:3 FLCTP1L/CBD heterotetramer, and 2:2 FLCTP1L/CBD heterotetramer. CBD is represented by *filled circles*, and the full-length (*FL*) protein is shown by *filled juggling clubs. B*, ESI-MS spectrum of the CTP1L CBDHis construct showing mostly monomers (10.32 kDa) and some dimers (20.65 kDa). *C*, ESI-MS spectrum of the CTP1L RBSKO mutant, which knocks out the internal ribosomal binding site, showing exclusively monomers. *D*, ESI-MS analysis of the knock-in silent mutant C → G for the ribosomal binding site of CTP1L, showing the presence of heterodimers and heterotetramers. *E*, the *inset* shows *bar graphs* of the relative abundance of reformed complexes containing RBSKO CTP1L mutant species at equimolar (1:1), 1:2, 1:3, and 1:6 molar excess of CTP1L CBDHis compared with the complexes formed of the CTP1L WT, determined from simulations of the raw data using Massign (from *left* to *right*: FL monomer, heterodimer, 1:3 heterotetramer, and 2:2 heterotetramer; CBDHis-only complexes are excluded for clarity). *Error bars*, S.D.

The rescue of lytic activity by the addition of increasing amounts of CBD raises the question how the oligomerization of full-length endolysin is affected by the truncated CBD. Native MS was used to analyze the oligomerization of the intact proteins (41, 42) to provide a semiquantitative snapshot of protein complexes as they occur in solution (43). Wild type CTP1L endolysin was found to predominantly exist as a heterotetramer (Fig. 6*A*), confirming the SAXS analysis in solution. The mass spectrum showed that ∼70% of the detected signals belonged to a heterotetramer with 2:2 stoichiometry, suggesting that it is the main active component (Table 4). Other oligomeric species present included a heterotetramer consisting of three copies of the CBD and one copy of the full-length CTP1L, a heterodimer, and a CBD-only homotetramer. The CBDHis alone formed both monomers and dimers in solution (Fig. 6*B*). Notably, the CTP1L RBSKO mutant, which knocks out the secondary translation site, only formed monomers (Fig. 6*C*). This indicates that for CTP1L, the full-length endolysin cannot oligomerize when the truncated CBD is not present, possibly due to steric hindrance by the catalytic domain. The C → G reverse mutation that restores part of the RBS and CBD production formed the same complexes as wild type CTP1L, including the heterodimer and heterotetramer (Fig. 6*D*). The monomeric form of the full-length CTP1L was also present in high amounts for the C → G mutation. Potentially, the lower CBD production led to a relative excess of full-length CTP1L, unassociated with CBDs (Table 4).

**TABLE 4:** **Ratios determined via simulation of MS peaks from full-length CTP1L monomers and CBDHis subunits from three independent measurements** The protein species containing only CBD (monomer, homodimers, and homotetramers) were omitted for clarity. Values are means ± S.D. Rounding to significant digits accounts for deviations to 1.0 in total. NA, not applicable.

Protein species	1:1 ratio	1:2 ratio	1:3 ratio	1:6 ratio	CTPL1 WT ratio	CTPL1 C→G ratio
FLCTP1L monomer	0.4 ± 0.2	0.20 ± 0.04	0.20 ± 0.03	0.14 ± 0.02	0.03 ± 0.02	0.28 ± 0.05
FLCTP1L: 1 CBD/CBDHis heterodimer	0.26 ± 0.01	0.29 ± 0.03	0.27 ± 0.01	0.31 ± 0.02	0.12 ± 0.02	0.09 ± 0.02
1 FLCTP1L: 3 CBD/CBDHis heterotetramer	0.09 ± 0.01	0.19 ± 0.03	0.27 ± 0.05	0.38 ± 0.02	0.14 ± 0.02	NA
2 FLCTP1L: 2 CBD/CBDHis heterotetramer	0.3 ± 0.2	0.32 ± 0.02	0.26 ± 0.02	0.17 ± 0.04	0.72 ± 0.04	0.62 ± 0.05

**FIGURE 7.**
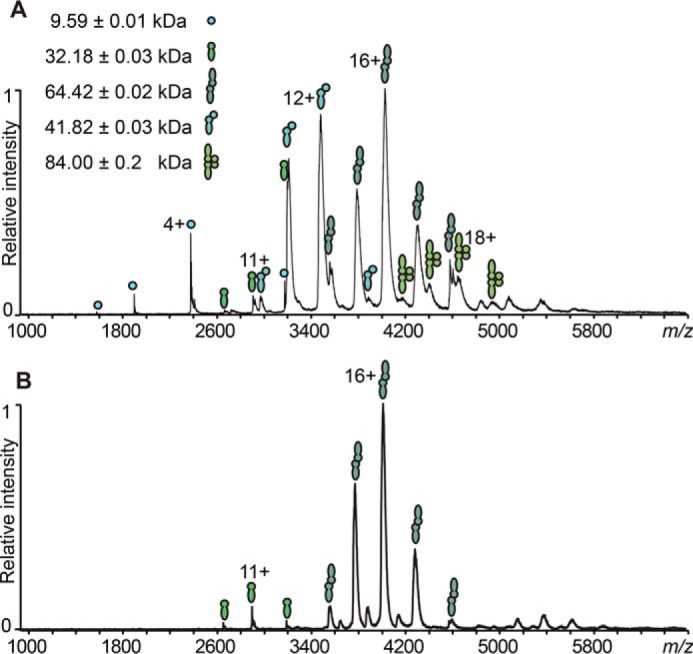
**ESI-MS comparison of the oligomerization occurring in CD27L wild type (*A*) and CD27L RBSKO mutant endolysin (*B*).** *Filled circles*, CBD; *filled juggling clubs*, full-length protein. The assigned protein species are CBD monomer, FLCD27L monomer, FLCD27L homodimer, 1:1 FLCD27L/CBD heterodimer, and 2:2 FLCD27L/CBD heterotetramer.

A different picture emerged for CD27L, which formed predominantly homo- and heterodimers and only little heterotetramer according to native MS analysis (Fig. 7*A*). This is in line with previous SAXS and static light scattering experiments in solution, where the homodimer was found to be predominant (16). When the ribosomal binding site is knocked out with the E180S/G181S mutation, the homodimer of the full-length endolysin was still formed in contrast to the CTP1L mutant (Fig. 7*B*).

To further analyze the effect that the truncated CBD has on the oligomeric state of the full-length CTP1L endolysin and to identify the active components, CBDHis was titrated in different ratios to the CTP1L RBSKO mutant (Figs. 6*E* (*inset*) and 8). At a ratio of 1:1, almost all of the truncated CBD had formed a complex with full-length CTP1L. The predominant oligomers were the heterodimer (26%) and the 2:2 heterotetramer (30%). At larger CBD ratios (1:2 and 1:3), more heterotetramers that contain one copy of the full-length CTP1L endolysin were formed, whereas the population of heterodimer and 2:2 heterotetramer stayed relatively constant. The distribution of species in the wild type was never reached. In view of the lytic assays presented in Fig. 5, *B* and *C*, this suggests that both heterotetramers contribute to the overall lytic activity. There was also an increase in free CBD, which could potentially compete with the active endolysin for binding epitopes. Overall, this titration experiment provides further evidence that the oligomerization driven by the truncated CBD regulates the activity of CTP1L endolysin.

**FIGURE 8.**
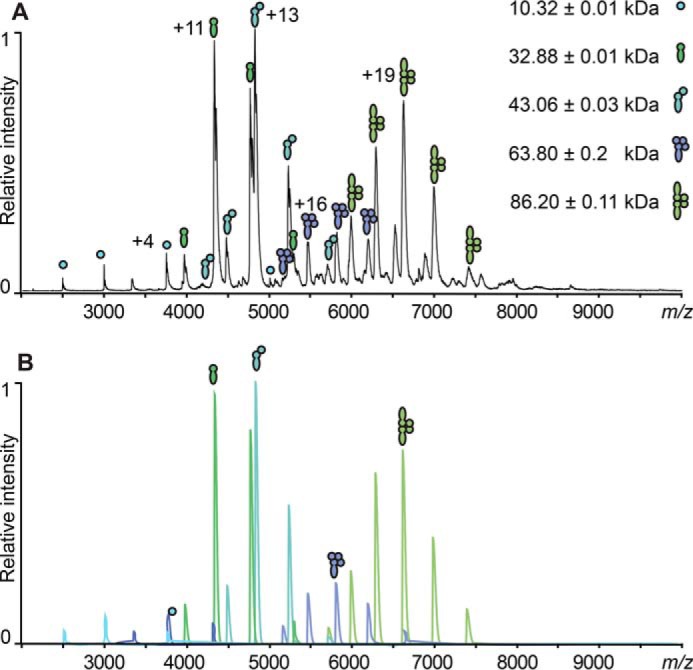
*A*, original spectra and exemplified Massign simulations of the formed complexes to determine complex ratios; *top*, representative spectrum of the reformed complexes containing RBSKO CTP1L mutant species equimolar (1:1) to CBDHis with the assigned complexes of CBDHis, FL monomer, FL heterodimer, 1:3 heterotetramer, and 2:2 heterotetramer. *Filled circles*, CBD; *filled juggling clubs*, full-length protein. *B*, the spectra shown were simulated for each protein species assigned in the original spectrum. The peak simulations were used to determine the percentages of the protein complexes by comparing their integrated peak area.

## Discussion

The crystal structure of the activated CTP1L endolysin presented here shows that an oligomer is formed between the full-length endolysin and the product of a secondary translation site within the endolysin gene, a truncated CBD. The truncated CBD mainly interacts with the CBD of the full-length endolysin (Fig. 1). The active site of the N-terminal glycosyl hydrolase domain is far away and is not affected by the binding of the truncated CBD. Previously, we have determined that the CBD plays an essential role in CTP1L activation through an oligomeric switch between two dimeric states (16). The two dimerization modes that we observe in the crystal structure of the activated CTP1L endolysin are identical to those observed for the CBDs alone. Mutagenesis disrupting the dimerization mode between full-length CTP1L and the truncated CBD leads to complete inactivation of CTP1L along the so-called side-by-side dimer (16).

Initially, we pursued the idea that the isolated C-terminal domains that crystallized on their own were the product of autoproteolytic cleavage. The identification of N-terminal methionines and an alternative start codon suggests that this is not the case but that the isolated domains are separate protein translation products. However, we still consider that autocleavage is possibly part of the endolysin mechanism. It should be noted that the electron density of the linker between the catalytic domain and the CBD is rather weak, suggesting that it may undergo cleavage. This is further corroborated by the observation that the crystals of the heterotetramer CTP1L deteriorate quickly and dissolve within 2 weeks. The presence of a sulfur atom in the dimer interface at the very N terminus of the truncated CBD suggested that it could play a role in autoproteolysis. The methionine side chain at position 195 of the truncated CBD is only 3.5 Å away from the Val-195 side chain of the endolysin (Fig. 3*C*). In the present study, we show that shutting down the secondary translation site reduces but does not fully abolish lysis. However, disruption of the side-by-side dimer, which is essential to bring the N-terminal methionine of the truncated CBD close to the linker of the full-length endolysin, completely inactivates the endolysin. It is therefore possible that other factors contribute to endolysin activity, and this probably involves interactions with components of the host cell wall.

The full-length endolysin can still dimerize through a head-on dimerization mode in high concentrations, although this is not observed for CTP1L in the native mass spectrometry experiments at relatively low concentrations. Interestingly, the CD27L endolysin is detected as a homodimer *in vacuo*. The lysis activity of this endolysin is not inhibited when one of the dimerization modes is blocked by mutagenesis, and the catalytic domain alone is in fact more active than the full-length endolysin when applied externally (5).

It therefore seems likely that the CBD oligomer forms a platform that binds to components of the cell wall to assist in the binding and exposure of the substrate of the catalytic domain. There is an interesting analogy with the structural organization of the PlyC endolysin, which contains an octameric assembly of the cell wall binding domain (12). This domain is encoded by a separate gene but on the same operon as the catalytic domain, and controlled variation of the expression of this domain may also regulate endolysin activity. We show that incubation of CTP1L lacking the secondary translation site with free CBD tunes lysis efficiency. At lower endolysin concentrations, the lysis efficiency increases with the addition of free CBD. At higher concentrations, the lysis efficiency decreases again. This is probably the result of the binding of free CBD to cell binding epitopes, preventing access for the full-length endolysin.

Alternative start sites have been described in several phages, notably the holin-antiholin system in phage λ (44), and there are several examples of “in-phase” gene overlapping (45). With regard to lysins, two overlapping genes within a lysin of *L. lactis* have been reported (46). The use of a secondary translation site as an oligomeric switch may be widespread among bacteriophages that target Gram-positive bacteria. Bacteriophages make very economical use of their genetic material, and the holin and endolysin genes are often overlapping. In-frame secondary translation has previously been reported for the staphylococcal phage 2638A, which is lytic for *Staphylococcus aureus* (47). In this case, the endolysin consists of two enzymatic units and a regulatory or cell wall binding domain. The secondary translation occurs between the two enzymatic domains and heightens endolysin activity. Recently, it was reported that the endolysin gene *lys170* also contains an internal translation start site that leads to the production of the C-terminal domain (48). In this paper, we show that the expression of a truncated protein leads to the formation of protein complexes with differing activities. Similar mechanisms may also apply to the coordinated regulation of the production of the holin and the endolysin (46), and it will be of great interest to investigate whether direct protein-protein interactions between holins and endolysins occur.

The transformation of the *L. lactis* strain with the CTP1L endolysin showed that the secondary translation product is also produced in commensal bacteria, and the fact that it was co-purified by affinity chromatography with the His-tagged full-length endolysin suggests that oligomerization also occurred. Continuous delivery of endolysins to the same environment as the target could combat problems of proteolysis, and the use of lactic acid bacteria has shown potential (18). Milk fermentation is affected by *C. tyrobutyricum* colonization, and the use of a recombinant *L. lactis* strain that secretes endolysins to eradicate *Clostridium* colonization during fermentation holds great promise. However, the successful implementation of this approach has been limited so far. Endolysin exported by a signal peptide may not achieve the oligomeric conformation for optimal activity, and this study highlights the need to design delivery systems to ensure that the most effective oligomeric structures are formed to improve biocontrol and therapeutic potential (*e.g.* by co-exporting separate CBDs or by designing genes with a second CBD attached to a flexible linker).

## Author Contributions

M. D. designed and performed experiments, produced all protein samples, and contributed to the writing of the manuscript. S. L. and J. K. performed mass spectrometry analysis on the truncated translation product. B. K. and C. U. performed and analyzed the native mass spectrometry experiments. H. D. T. M. and D. I. S. performed and analyzed SAXS experiments. A. T. assisted in the determination and interpretation of the x-ray crystal structures. N. G.-T. and S. G. performed and analyzed the experiments on the cell wall binding assays together with A. N. and M. J. M. M. J. M. performed and analyzed the lysis assays and the *L. lactis* work and contributed to the design of experiments and the writing of the manuscript. R. M. conceived and led the project and wrote the manuscript with input from all authors.

## Supplementary Material

Supplemental Data

## Footnotes

4 The abbreviations used are:

SAR: signal arrest release
CBD: C-terminal cell wall binding domain
sCTP1L: synthetic CTP1L
Ni-NTA: nickel-nitrilotriacetic acid
SAXS: small angle x-ray scattering
ESI: electrospray ionization
RBSKO: ribosomal binding site knock-out.
