# Supplementary material for: Crystal Structure of the CTP1L Endolysin Reveals How Its Activity Is Regulated by a Secondary Translation Product
Source: J Biol Chem. 2015 Dec 18;291(10):4882–93. doi: 10.1074/jbc.M115.671172 (PMC4777826; doi:10.1074/jbc.M115.671172)
Supplement: Supplemental Data [file supp_291_10_4882__index.html]

Crystal structure of the CTP1L endolysin reveals how its activity is regulated by a secondary translation product — Crystal Structure of the CTP1L Endolysin Reveals How Its Activity Is Regulated by a Secondary Translation Product — CTP1L Endolysin Regulated by a Secondary Translation Product — Supplemental Data 

# Crystal Structure of the CTP1L Endolysin Reveals How Its Activity Is Regulated by a Secondary Translation Product

## Supplemental Data

- Supplementary Figure S1 (.pdf, 2.2 MB) - Upon request of the reviewers, this file should be added as a supplement.
