# Supplementary material for: Crystal Structure of the CTP1L Endolysin Reveals How Its Activity Is Regulated by a Secondary Translation Product
Source: J Biol Chem. 2015 Dec 18;291(10):4882–93. doi: 10.1074/jbc.M115.671172 (PMC4777826; doi:10.1074/jbc.M115.671172)
Supplement: Supplemental Data [file 10.1074_M115.671172_jbc.M115.671172-1.pdf]

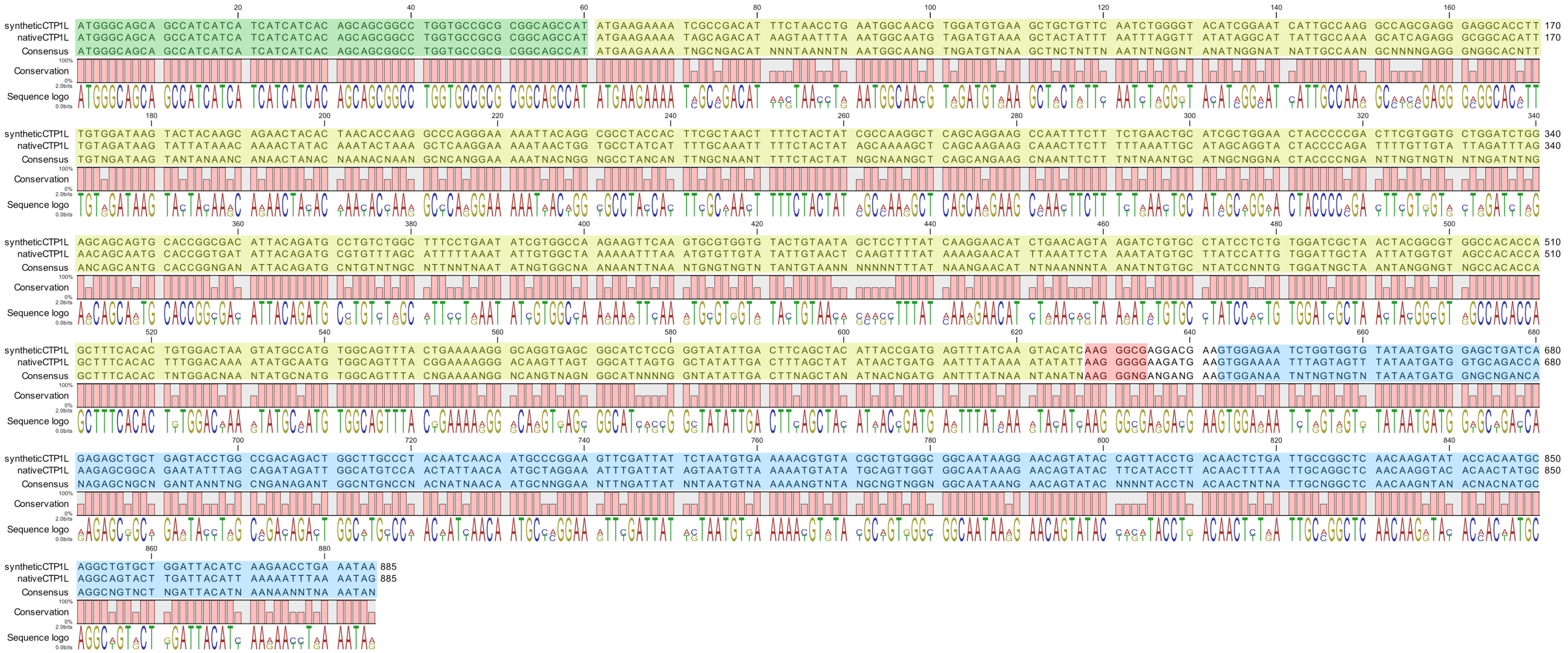

Supplementary figure S1. Alignment of both the full-length native CTP1L and synthetic CTP1L nucleotide sequences. In green (1-80) is the conserved N-terminal His-tag and thrombin cleavage site, in yellow (81-640) the Catalytic domain, in red the putative ribosomal binding site (628-634), where the single nucleotide change G > C was produced upon generation of synthetic CTP1L. In blue the cell wall binding domain (643-885) is shown starting with the GTG alternative start codon. Alignment was generated using CLC Genomics workbench version 7.5.1.
